# Robot-assisted pancreatoduodenectomy with preservation of the vascular supply for autologous islet cell isolation and transplantation: a case report

**DOI:** 10.1186/1752-1947-6-74

**Published:** 2012-03-02

**Authors:** Piero Giulianotti, Veronica Gorodner, Katie Kinzer, Enrico Benedetti, Jose Oberholzer

**Affiliations:** 1 Division of Surgery/Transplant, University of Illinois at Chicago, 840 South Wood Street CSB, Chicago IL 60612, USA; 2 Ruta 58, Km 8, Barrio Campo Azul, Lote 120, Canning, Esteban Echeverria CP 1804, Buenos Aires, Argentina

## Abstract

**Introduction:**

For patients with chronic pancreatitis presenting with medically intractable abdominal pain, surgical intervention may be the only treatment option. However, extensive pancreatic resections are typically performed open and are associated with a substantial amount of postoperative pain, wound complications and long recovery time. Minimally invasive surgery offers an avenue to improve results; however, current limitations of laparoscopic surgery render its application in the setting of chronic pancreatitis technically demanding. Additionally, pancreatic resections are associated with a high incidence of diabetes. Transplantation of islets isolated from the resected pancreas portion offers a way to prevent post-surgical diabetes; however, preservation of the vascular supply during pancreatic resection, which determines islet cell viability, is technically difficult using current laparoscopic approaches. With recent advances in the surgical field, robotic surgery now provides a means to overcome these obstacles to achieve the end goals of pain relief and preserved endocrine function. We present the first report of a novel, minimally invasive robotic approach for resection of the pancreatic head that preserves vascular supply and enables the isolation of a high yield of viable islets for transplantation.

**Case presentation:**

A 35-year old Caucasian woman presented with intractable chronic abdominal pain secondary to chronic pancreatitis, with a stricture of her main pancreatic duct at the level of the ampulla of Vater and distal dilatation. She was offered a robotic-assisted pylorus-preserving pancreatoduodenectomy and subsequent islet transplantation, to both provide pain relief and preserve insulin-secretory reserves.

**Conclusion:**

We present a novel, minimally invasive robotic approach for resection of the pancreatic head with complete preservation of the vascular supply, minimal warm ischemia time (less than three minutes) and excellent islet recovery (134,727 islet equivalent). Our patient is currently pain-free with normal glycemic control. Robot-assisted pylorus-preserving pancreatoduodenectomy and autologous islet transplantation can be safely performed and has the potential to minimize operative traumas as well as to partially preserve endocrine function. Results from this case report suggest that this dual procedure should be considered as a treatment option for patients with chronic pancreatitis at earlier stages of the disease, before irreversible islet loss occurs.

## Introduction

Chronic pancreatitis (CP) is an inflammatory process of the pancreas characterized by irreversible morphological changes that can lead to impaired endocrine and exocrine function. The treatment of patients with CP remains controversial; conservative treatment is typically preferred over surgery. Surgical intervention is often considered late in the course of the disease and remains the last resort for patients presenting with medically intractable abdominal pain [1]. In these cases, treatment needs to be adapted to the individual situation, as CP is a heterogeneous group of diseases with different etiologies and presentations. Several aspects must be considered, such as the surgical approach, metabolic consequences and surgical technique. For instance, decompression of the pancreatic duct may be the most appropriate treatment if the pancreatic duct is dilated, whereas partial or total pancreatic resection may be the only option if the pancreatic duct is normal in size or narrow. In patients whose symptoms are not resolved after drainage procedures or partial pancreatic resections, or those who have diffuse involvement of the gland, a total pancreatectomy may be the only treatment option [2].

While total pancreatectomy classically provides pain relief in about 70% of patients [2], it is not an innocuous procedure. Extensive pancreatic resections are typically performed open and are associated with a substantial amount of postoperative pain, wound complications and long recovery time. While this has motivated several attempts to adopt a minimally invasive approach, laparoscopic pancreatic surgery persists as one of the most challenging applications of minimally invasive surgery. Since the first report of laparoscopic pylorus-preserving pancreatoduodenectomy in 1994, further attempts have failed to report significant benefits over an open approach [3,4]. A recent literature review of 146 procedures since 1994 found that a laparoscopic approach is not universally accepted due to the technical difficulty for the surgeon, length of operating time and the absence of a reduced length of hospital stay for the patient [5].

Recently, surgeons have begun to overcome the limitations of traditional laparoscopic surgery through robotic surgery. The robotic system improves the performance of minimally invasive surgery by restoring three-dimensional vision, enhancing surgeon dexterity and eliminating tremor. Recently, we published the largest series of robotic pancreatic surgeries (n = 124) to date, demonstrating both feasibility and safety [6]. Additional attempts have demonstrated the success of robot-assisted pancreatic resections and reconstructions [7].

Although the main goal of the operation is to improve the quality of life in patients with CP by providing pain relief, metabolic consequences should not be ignored. Post-pancreatectomy diabetes is typically brittle, as a result of concomitant deficiency in insulin secretion and counter regulatory hormones, and difficult to manage. Continued alcohol abuse or poor nutrition, combined with maldigestion, further complicate this management. It has been found that Whipple procedures (the standard pancreatoduodenectomy) result in a 20% increase in the incidence of diabetes [1]. In patients seeking surgical treatment for CP, the cost of long-term morbidity from diabetes and endocrine deficiency must be assessed.

Autologous islet transplantation (AIT), first reported to preserve islet function following a near-total pancreatectomy in 1977, has been consistently demonstrated as a safe procedure that can prevent diabetes long-term in patients with CP [8-10]. The success rate largely depends on the amount of islets infused and their viability, which is directly linked to the extent of ischemia they are exposed to during the pancreatectomy [9]. In order to avoid extended warm ischemia time, the vascular supply of the pancreas needs to be preserved until the last moment of the resection. Preservation of the vascular supply is difficult during a standard Whipple procedure. Additionally, the insulin-secretory reserves of the pancreatic remnant are typically sufficient to prevent postsurgical diabetes. Therefore, most surgeons would not consider isolating islets from solely the pancreatic head. However, the fate of the pancreatic remnant is unknown. Progressive fibrosis may destroy the remaining islets over time or lead to pain recurrence, necessitating a complete pancreatectomy. Furthermore, the probability of insulin independence after AIT progressively declines with increasing fibrosis [8]. Thus, it would be beneficial to preserve islets during the initial pancreatic head resection.

In addition to providing a platform to perform a minimally invasive pancreatectomy in the technically demanding setting of CP, robot assistance offers the possibility to preserve the vascular supply of the pancreatic head during surgery for islet isolation. In consideration of the high rate of diabetes (approximately 50% five years after onset) observed in the natural history of CP [11,12], surgical intervention that successfully provides pain relief and preserves the endocrine function earlier in the course of CP would be of significant benefit to the patient. Herein we present a case report documenting our experience with robot-assisted pylorus-preserving pancreatoduodenectomy (RA-PPPD) with minimal warm ischemia time and excellent islet recovery for AIT in a patient with CP.

## Case presentation

Our patient was a 35-year old Caucasian woman with CP of unknown etiology. Her main complaint at the time of the consultation was severe chronic abdominal pain refractory to narcotic pain medication. Additionally, she was experiencing nausea and vomiting. Her pancreatic function was normal, with a hemoglobin A1C level of 5.4% and basal C-peptide level of 1.3 ng/mL. The history of her present illness was significant for several episodes of recurrent pancreatitis over the past six years after undergoing an open cholecystectomy with subsequent removal of a retained common bile duct stone by endoscopic retrograde cholangiopancreatography (ERCP). Her past medical history was also significant for psoriasis and heavy alcohol consumption, which likely contributed to the subsequent development of CP, although she reported abstinence for the past year.

At the time of her admission, an ERCP was performed and demonstrated the presence of a previous sphincterotomy and a dilated common bile duct of approximately 11 mm without filling defects. A stricture of her main pancreatic duct was observed at the level of the ampulla of Vater; dilatation of the remaining portion of the pancreatic duct was present. An endoscopic ultrasound was performed and revealed sonographic changes consistent with mild CP (2.5 mm pancreatic duct, heterogeneous parenchyma) according to the Cambridge classification [13]. Magnetic resonance cholangiopancreatography and computed tomography of her abdomen were also performed and demonstrated findings consistent with those observed in the ERCP. Given the young age of our patient, we gave priority to surgical therapy to avoid additional endoscopic procedures. After discussing the various therapeutic options with our patient, she chose an RA-PPPD, for better drainage of the distal pancreas and pain relief. Additionally, in consideration of her young age and the future unknown fate of the pancreatic remnant, our patient was offered AIT to preserve endocrine function.

**Figure 1:**
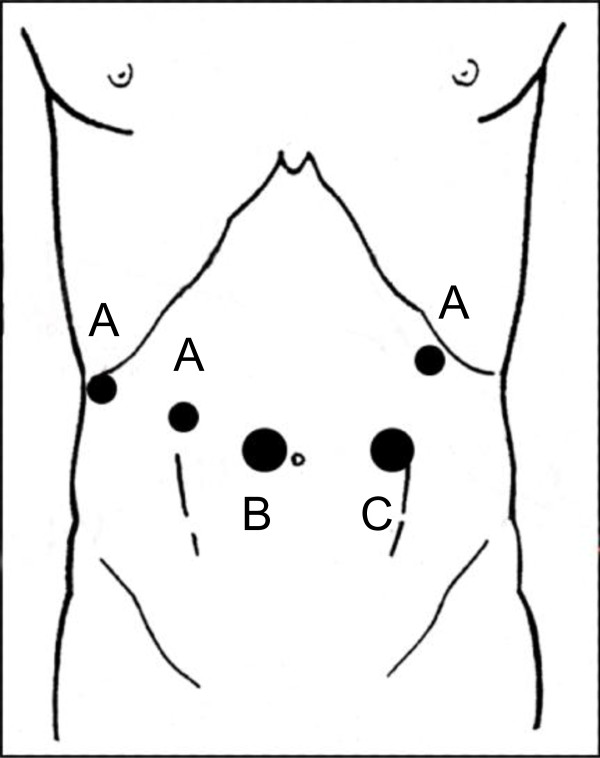
**Trocar Positioning**. **(A) **Three 7 mm trocars, for the robotic arms, were inserted - two on her right flank and one in the upper left quadrant. **(B) **One 12 mm trocar, for the robotic scope, was inserted towards the right of her umbilicus. **(C) **Another 12 mm trocar, for the assistant, was inserted toward the left side of her umbilicus.

**Figure 2:**
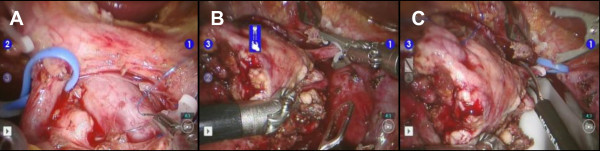
**Resection of the pancreatic head**. **(A) **The gastroduodenal artery (GDA) was prepared and a vessel loop was placed. Once the pancreatic head was completely mobilized, the GDA was **(B) **ligated and **(C) **transected, with immediate removal of the specimen for cold flush with preservation solution on the back-bench.

After induction of general anesthesia, our patient was placed in the lithotomy position, with slight reverse Trendelenburg, and her abdomen was prepared and draped in the usual sterile fashion. Trocars were placed as indicated in Figure 1. The Da Vinci robotic surgical system (Intuitive Surgical, Inc. Sunnyvale, CA, USA) was docked into position, with a viewpoint from our patient's head. We mobilized the right colonic flexure, exposed the second portion of her duodenum and completed mobilization of the pancreatic head. The pancreatic head was enlarged and fibrotic. Next, her hepatic hilum was dissected, her common hepatic artery was exposed and her right gastric artery was ligated. The origin of her gastroduodenal artery was prepared with a vessel loop but not divided (Figure 2A) to preserve the blood supply to the head of the pancreas. The dissection of her gastrocolic ligament was completed, exposing the inferior border of her pancreas and the pancreatic neck. The neck of her pancreas was prepared and her superior mesenteric vein was widely exposed. Next, the pylorus was prepared; her right gastroepiploic artery and vein were divided. The first portion of her duodenum was divided 2 cm distal to the pylorus using a stapling device. The first loop of her jejunum was transected using a stapler device as well. Her duodenum was retracted, exposing the uncinate process. Subsequently, her common bile duct was transected and the dissection was conducted in the neck of her pancreas, dividing the pancreatic neck with the Harmonic scalpel. Her pancreatic duct was enlarged, measuring approximately 3 mm to 4 mm. The dissection proceeded cautiously until the entire head of her pancreas was connected only to her gastroduodenal artery and superior pancreaticoduodenal vein. Immediately before the transection, a small Pfannenstiel incision was made; a hand access device (Lap Disc, Ethicon, Cincinnati, OH, USA) was inserted with the aim of preserving the pneumoperitoneum. At this point, her gastroduodenal artery and superior pancreaticoduodenal vein were clipped and divided (Figure 2B, C). The specimen was placed in an endobag and extracted immediately through the mini laparotomy previously performed. Her pancreas was flushed with University of Wisconsin solution on the back-bench and brought to the islet isolation facility in a sterile bag on ice for processing.

In the interim, the reconstruction phase of the operation was initiated. A pancreaticogastrostomy was performed. Next, a retrocolic end-to-side hepaticojejunostomy was created with the first loop of her jejunum. The last anastomosis was an end-to-side two layer pylorojejunostomy, 40 cm distal to the hepaticojejunal anastomosis. Once the reconstruction was completed, her inferior mesenteric vein was dissected and canulated with a 16-gauge canula in preparation for the islet cell infusion.

**Figure 3:**
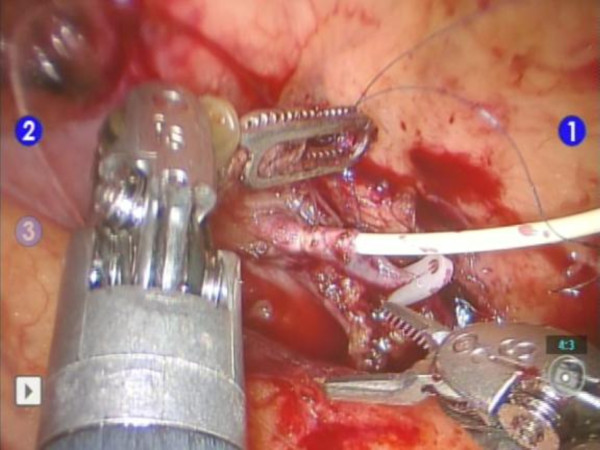
**Ligation of her inferior mesenteric vein**. Her inferior mesenteric vein was identified, dissected and distally ligated. A canula was inserted for infusion of the pancreatic digest for autologous islet transplantation.

The islet isolation procedure was conducted as previously described [14]. In brief, the pancreatic duct was canulated and injected with a purified collagenase solution (Serva, Heidelberg, Germany). The pancreas was cut into small pieces and placed into a modified Ricordi digestion system. Under microscopic control of repeat samples, the digestion was stopped by dilution and cooling as soon as 50% of the islets were free from the exocrine tissue. The digest was collected and washed under repeat centrifugation. After a quality assessment the islet preparation was placed into several syringes and brought into the operating room. The islets were successfully infused into the portal stream and her inferior mesenteric vein was subsequently ligated (Figure 3). To conclude, two Jackson Pratt #10 drainages were placed - one adjacent to the pancreatic anastomosis and one underneath her liver.

Our patient tolerated the procedure well. The weight of the resected pancreas portion was 47 g, after trimming of non-pancreatic tissue. Fifteen milliliters of tissue containing 134,727 islet equivalent (IEQ; 2867 IEQ per gram of pancreas or 2449 IEQ per kilogram of the recipient body weight) were collected. The viability of the tissue was 97%, as measured by propidium iodide and cytogreen fluorescent staining. The operation lasted six hours and thirty minutes, with an estimated blood loss of 200 mL. Our patient was maintained on an intravenous insulin drip during the first two days after the operation. She was subsequently transitioned to 4U to 6U of long-acting insulin daily. Our patient's recovery was uneventful; her C-peptide level on the eighth postoperative day was 2.2 ng/mL. She was discharged home on the ninth postoperative day with pain improvement and was maintained on low dose insulin. During her last follow-up 45 days after the surgery, she was normoglycemic without any insulin injection and reported complete resolution of her pain.

## Discussion

In this case, we were able to preserve the vascular supply to the pancreatic head until the conclusion of the procedure, ligating the gastroduodenal artery immediately before transecting the head of the pancreas. This was facilitated by a view and approach, provided by the position of the robotic arms and three-dimensional camera, that is difficult and only rarely possible in open surgery. The warm ischemia time was under three minutes and we were able to isolate 2449 IEQ/kg, which is a remarkable islet yield considering that only the pancreatic head was resected. The chances of remaining insulin independent are significantly increased if the islet yield is above 2500 IEQ/kg [8-10].

## Conclusion

We present a novel, minimally invasive robotic approach for resection of the pancreatic head with complete preservation of the vascular supply, minimal warm ischemia time and excellent islet recovery. RA-PPPD and AIT can be safely performed, with the potential to minimize operative trauma and partially preserve endocrine function. Transplantation of a high yield of viable islets from the resected pancreatic portion in our patient reduces the risk of her developing diabetes in the future. Pancreatic resection for CP has good long-term efficacy for pain control, but is typically performed late in the course of the disease. With the availability of RA-PPPD and AIT, surgical intervention should be considered and offered earlier, to preserve endocrine function before irreversible islet loss.

## Consent

Written informed consent was obtained from the patient for publication of this case report and any accompanying images. A copy of the written consent is available for review by the Editor-in-Chief of this journal.

## Competing interests

The authors declare that they have no competing interests.

## Authors' contributions

PG, VG, EB and JO were major contributors in the patient diagnosis, treatment decision, surgical performance and patient follow-up. VG also contributed significantly to the manuscript writing. KK was a major contributor in patient follow-up, the literature research and manuscript writing. All authors read and approved the final manuscript.

## Authors' information

PG holds the title of Lloyd M. Nyhus Endowed Chair in Surgery, Chief of the Division of General, Minimally Invasive and Robotic Surgery at University of Illinois at Chicago. EB holds the title of Warren H. Cole Chair in Surgery, Professor and Head of the Department of Surgery at University of Illinois at Chicago. JO holds the title of Chief of the Division of Transplantation, as well as Director of the Islet and Pancreas Transplant Program, and Professor of Surgery, Bioengineering and Endocrinology at University of Illinois at Chicago.

## References

[B1] Evans JD Wilson PG Carver C Bramhall SR Buckels JA Mayer AD McMaster P Neoptolemos JP Outcome of surgery for chronic pancreatitis Br J Surg 1997 84 624 629 10.1002/bjs.1800840512 9171747

[B2] Talamini G Bassi C Butturini G Falconi M Casetti L Gumbs AA Carrara S Fantin A Pederzoli P Outcome and quality of life in chronic pancreatitis Jop 2001 2 117 123 11875248

[B3] Gagner M Pomp A Laparoscopic pylorus-preserving pancreatoduodenectomy Surg Endosc 1994 8 408 410 10.1007/BF00642443 7915434

[B4] Gagner M Pomp A Laparoscopic pancreatic resection: Is it worthwhile? J Gastrointest Surg 1997 1 20 25 discussion 25-26 10.1007/s11605-006-0005-y 9834326

[B5] Gagner M Palermo M Laparoscopic Whipple procedure: review of the literature J Hepatobiliary Pancreat Surg 2009 16 6 726 730 10.1007/s00534-009-0142-2 19636494

[B6] Giulianotti PC Sbrana F Bianco FM Elli EF Shah G Addeo P Caravaglios G Coratti A Robot-assisted laparoscopic pancreatic surgery: single-surgeon experience Surg Endosc 2010 24 1646 1657 10.1007/s00464-009-0825-4 20063016

[B7] Zureikat AH Nguyen KT Bartlett DL Zeh HJ Moser AJ Robotic-assisted major pancreatic resection and reconstruction Arch Surg 2010 10.1001/archsurg.2010.246 21079111

[B8] Wahoff DC Papalois BE Najarian JS Kendall DM Farney AC Leone JP Jessurun J Dunn DL Robertson RP Sutherland DE Autologous islet transplantation to prevent diabetes after pancreatic resection Ann Surg 1995 222 562 575 discussion 575-569 7574935 10.1097/00000658-199522240-00013 PMC1234892

[B9] Oberholzer J Triponez F Mage R Andereggen E Buhler L Cretin N Fournier B Goumaz C Lou J Philippe J Morel P Human islet transplantation: lessons from 13 autologous and 13 allogeneic transplantations Transplantation 2000 69 1115 1123 10.1097/00007890-200003270-00016 10762216

[B10] Anazawa T Balamurugan AN Bellin M Zhang HJ Matsumoto S Yonekawa Y Tanaka T Loganathan G Papas KK Beilman GJ Hering BJ Sutherland DE Human islet isolation for autologous transplantation: comparison of yield and function using SERVA/Nordmark versus Roche enzymes Am J Transplant 2009 9 2383 2391 10.1111/j.1600-6143.2009.02765.x 19663895 PMC7652598

[B11] Berney T Rudisuhli T Oberholzer J Caulfield A Morel P Long-term metabolic results after pancreatic resection for severe chronic pancreatitis Arch Surg 2000 135 1106 1111 10.1001/archsurg.135.9.1106 10982519

[B12] Ito T Otsuki M Itoi T Shimosegawa T Funakoshi A Shiratori K Naruse S Kuroda Y Pancreatic diabetes in a follow-up survey of chronic pancreatitis in Japan J Gastroenterol 2007 42 291 297 10.1007/s00535-006-1996-6 17464458

[B13] Banks PA Classification and diagnosis of chronic pancreatitis J Gastroenterol 2007 42 Suppl 17 148 151 17238045 10.1007/s00535-006-1922-y

[B14] Giulianotti PC Kuechle J Salehi P Gorodner V Galvani C Benedetti E Oberholzer J Robotic-assisted laparoscopic distal pancreatectomy of a redo case combined with autologous islet transplantation for chronic pancreatitis Pancreas 2009 38 105 107 10.1097/MPA.0b013e31816b3100 19106750

